# Mutant *dlx3b* disturbs normal tooth mineralization and bone formation in zebrafish

**DOI:** 10.7717/peerj.8515

**Published:** 2020-02-19

**Authors:** Liping Pang, Zhichun Zhang, Yan Shen, Zhenchao Cheng, Xuejun Gao, Bo Zhang, Xiaoyan Wang, Hua Tian

**Affiliations:** 1 Department of Cariology and Endodontology & National Clinical Research Center for Oral Disease & Beijing Key Laboratory of Digital Stomatology, School and Hospital of Stomatology, Peking University, Beijing, PR China; 2 Key Laboratory of Cell Proliferation and Differentiation of the Ministry of Education, College of Life Sciences, Peking Universiy, Beijing, PR China

**Keywords:** dlx3b, Zebrafish, Tooth, Bone

## Abstract

**Background:**

Tricho-dento-osseous (TDO) syndrome is an autosomal dominant disorder characterized by anomalies in hair, teeth and bone (OMIM190320). Various mutations of Distal-Less 3 (*DLX3*) gene are found to be responsible for human TDO. The aim of this study was to investigate effects of *DLX3* on tooth and bone development using a zebrafish model.

**Methods:**

The *dlx3b* mutant zebrafish lines were established using the gene targeting tool transcription activator-like effector nuclease (TALEN). Micro-computed tomography was used to render the three-dimensional skeletal structures of mutant fishes. The pharyngeal bone along with connected teeth was isolated and stained by Alizarine Red S, then observed under stereomicroscope. Scanning electron microscopy (SEM) and energy dispersive spectrometer (EDS) were used to examine the tooth surface morphology and mineral composition. Quantitative real-time PCR was used to analyze gene expression.

**Results:**

A moderate curvature of the spine toward the dorsal side was found at the early larval stages, appearing in 86 out of 100 larvae in *dlx3b^-/-^* group as compared to 3 out of 99 in the *dlx3b^+/+^* group. At the adult stage, three of the thirty *dlx3b^-/-^* homozygotes exhibited prominent abnormal curvature in the spine. SEM revealed morphological surface changes in pharyngeal teeth enameloid, accompanied by a decrease in the mineral content detected by EDS. Furthermore, specific secretory calcium-binding phosphoprotein (SCPP) genes, including *odam*, *scpp9*, *spp1*, *scpp1*, and *scpp5* were significantly downregulated in *dlx3b* mutants.

**Conclusion:**

The findings of this study suggest that *dlx3b* is critical for enamel mineralization and bone formation in zebrafish. Moreover, the discovery of the downregulation of SCPP genes in *dlx3b* mutants sheds new light on the molecular mechanisms underlying TDO syndrome.

## Introduction

Mutation of the Distal-less homeobox 3 (*DLX3*) gene is responsible for the human tricho-dento-osseous syndrome (TDO; OMM 190320), which is characterized by abnormal hair, teeth and bone development. Multiple types of *DLX3* mutations have been reported in individuals with TDO. All of the mutations are located within or adjacent to the homeodomain of the DLX3 protein (Whitehouse et al., 2018). The most common dental anomalies of TDO include severe enamel hypoplasia, thin dentin, and taurodontism. Pitting or furrowing of the enamel surface, a typical feature of enamel hypoplasia, is common in TDO (Wright et al., 1997; Price et al., 1998). In addition to tooth defects, other primary phenotypic characteristics manifested in TDO cases include an increased thickness and density of cortical bone in many regions, including the spine, limbs, and craniofacial bones (Haldeman et al., 2004; Islam, Lurie & Reichenberger, 2005). TDO’s pleiotropic symptoms in both tooth and bone suggest that the *DLX3* gene might play an essential role in modulating the development of mineralized tissues.

Recently, the importance of *DLX3* has been shown using *in vitro* and *in vivo* models, although hypotheses about the underlying mechanisms are disparate and sometimes even conflicting. *In vitro* studies carried out by Zhao et al. (2016), using cell samples gathered from patients with TDO, indicated that DLX3 negatively regulated osteoblastic differentiation through microRNA-124. Choi et al. (2009) and Choi et al. (2010) had established a mutant *Dlx3* mouse model that carried the 2.3 *Col1a1* promoter. The mutant mouse exhibited enhanced differentiation in bone marrow cells and disrupted differentiation of odontoblasts, partially through the regulation of IFN-r and DSPP. Recently, using a *K14*-cre conditional *Dlx3*-deletion mouse model, the Morasso lab revealed that *Dlx3* affected ion transport in amelogenesis through the regulation of genes in the solute carrier (Slc) family (Duverger et al., 2016). In our previous *in vitro* study, we reported that DLX3 transactivated the expression of *Amelx*, *Enam,* and *Odam* in an ameloblast cell lineage (Zhang et al., 2015). From *Dlx3*’s various functions, we noted that several *Dlx3* downstream genes including *Dspp*, *Amelx*, *Enam,* and *Odam,* all belong to the secretory calcium-binding phosphoprotein (*SCPP*) gene family. All SCPP genes are derived from a common *SPARCL1* ancestor and are associated with bone and teeth mineralization (Kawasaki, 2009; Kawasaki, 2011). More studies and animal models are needed to clarify the exact function of *DLX3*, determine the primary mechanisms that cause TDO, and interpret the effects of *Dspp*, *Amelx*, *Enam*, and *Odam*, especially in tooth and bone formation (Daubert et al., 2016).

The zebrafish is widely used in mineralized tissue research due to its size and fecundity and because the genetic homology in early developmental process between zebrafish and human (Bartlett et al., 2005; Witten et al., 2017). The zebrafish *dlx3b* gene is conserved in human *DLX3* and exhibits spatiotemporal expression patterns in tooth and bone development (Borday-Birraux et al., 2006; Verreijdt et al., 2006). Notably, the expression of *dlx3b* was mainly identified in the epithelium of the tooth, which suggested that *dlx3b* might be involved in the differentiation and proliferation of dental epithelium in zebrafish (Borday-Birraux et al., 2006). Studies have reported that morpholino knockdown of *dlx3b* and other *dlx* members in zebrafish led to dramatic facial skeletal defects and misshaped teeth in larval fishes (Jackman & Stock, 2006; Talbot, Johnson & Kimmel, 2010). Nonetheless, the recent advent of efficient genome editing tools, such as TALEN and CRISPR/Cas9, has allowed for efficiently targeted deletions in selected genes of model zebrafish (Huang et al., 2016; DeLaurier, Alvarez & Wiggins, 2019). On the one hand, the altered gene function can be directly modelled by these zebrafish, which could provide insight into the cause of human TDO. On the other hand, a comparison of mutant phenotypes between human patients and zebrafish models might help to reveal the evolutionary functions of *DLX3* and *dlx3b*. Recently, CRISPR/Cas9-mediated deletion of *dlx3b* and *dlx4b* in zebrafish was proven to impair otic induction and the formation of sensory hair cells; however, the phenotype of the skeletal system was not mentioned (Schwarzer et al., 2017).

In this study, we generated a *de novo* mutation in the *dlx3b* gene that caused a frameshift change in the C-terminal sequence of the dlx3b homeodomain, which produced an early STOP codon after the homeobox. Morphological analysis and gene expression analysis were carried out in *dlx3b* ^−∕−^ zebrafish. We analyzed the shared essential roles of the *dlx3b* and the *DLX3* genes, focusing specifically on skeleton development, including the teeth and bone.

## Materials and Methods

### Animals and ethics statement

All zebrafish studies were conducted under the guidance and approval of the Institutional Animal Care and Use Committee at Peking University (LSC-ZhangB-1). Zebrafish embryos were incubated at 37 °C until 6 days post-fertilization (dpf). The water was changed daily. Larvae were fed with paramecium from 6–10 dpf and commercial baby fish food from day 10 onward. The adult fishes were maintained at 28.5 °C with a 14-h light/10-h dark cycle in a circulating system that continuously filtered, UV treated, and aerated the circulating water.

### Generation of the *dlx3b*^−∕−^ zebrafish line

TDO related DLX3 mutations are found to affect residues within, or adjacent to the homeodomain. The TALEN targeting site in the zebrafish *dlx3b* gene was designed at the 3-terminal end of the homeodomain, using TALE-NT (https://boglab.plp.iastate.edu/) and ZiFiT (http://zifit.partners.org/ZiFiT/). Then, the TALEN constructs were assembled using the unit assembly method (Huang et al., 2011). The TALEN mRNAs were synthesized using the SP6/T7 MESSAGE mMACHINE Kit, purified by LiCl precipitation, and injected into zebrafish embryos at the one-cell stage. The embryos were raised to adulthood and then outcrossed with wild-type fish to select founders carrying mutations. Mutations were further confirmed *via* HindIII digestion of PCR products and DNA sequencing. Two founders containing the targeted 4 bp deletion mutation were selected and outcrossed with wild type (WT) fish. The F1 embryos from the 2 selected founders were raised. Heterozygous F1 mutants were identified. Then, the F1 mutants were incrossed to breed enough F2 fish for subsequent experiments, including WT (*dlx3b*^+∕+^), heterozygous mutants (*dlx3b*^+∕−^), and homozygous mutants (*dlx3b*^−∕−^). The WT (*dlx3b*^+∕+^) were used as the control group. The number of specimens used in different experiments has been indicated in respective parts. All specimens used were identified using HindIII digestion of PCR products and DNA sequencing. The PCR primers used were: forward primer 5′-TTAGTGAGGCATTGTCGGCT-3′ and reverse primer 5′-CGCGGAGTATCTGTCAGCTT-3′. Zebrafish embryos at 6 dpf were allocated to three groups for further growth in separate dishes, starting with 100 embryos in each group. The dead fish were removed when replacing water. The number of surviving larval fish was counted and calculated from 6 to 10 dpf using Image J, and each experiment was repeated for three times.

### Scanning electron microscopy

Adult F2 zebrafish were collected when they were a standard length of 25 mm, approximately coinciding with 90 dpf. The pharyngeal jaws were carefully dissected under a stereomicroscope, immersed in 0.1% KOH for 48 h to remove the soft tissues, rinsed overnight with sterile water, and then freeze-dried for 4 h. The samples were fixed to a specific platform using conductive adhesive and coated with a palladium-gold film before scanning. The surface morphology of each sample was recorded using a scanning electron microscope (S4800, *n* = 10 for each group). Energy dispersive spectrometer analysis was then performed on a certain area of tooth enameloid using EMAX350 (*n* = 5 for each group).

### Micro-computed tomography analysis

To examine the three-dimensional structures of mineralized hard tissues, the adult zebrafishes were fixed in 4% paraformaldehyde for 48 h. The zebrafish samples were scanned using micro-CT (Inveon MM CT, SIEMENS, Munich, Germany) and then 3-D reconstructed using the NRecon (NRecon version 1.6.9.4, Skyscan, KONTICH, Belgium) software (*n* = 3 for each group). Eight-bit grayscale images, reconstructed using NRecon, were transferred to SkyScan^®^ CT analyser Version 1.13.5.1 (Kontich, Belgium). Three regions of interest (ROIs) were selected, and then the values for bone density from wide-type and homozygous mutants were calculated and analyzed.

### Real-time RT-PCR

Eight larvae at 96 h post-fertilization (hpf) were pooled for total RNA isolation of each group. Total RNA was isolated with a TRIzol reagent (Invitrogen, Waltham, CA, USA) according to the manufacturer’s instruction. The cDNA synthesis was carried out in a 10-µl reaction mixture containing 2 µg total RNA, 400 mM reverse transcription primers, 4 U/µl M-MLV, 1 U/µl RNAsin, and 0.4 mM dNTP mix, using M-MLV reverse transcriptase (Promega, Madison, WI, USA). The amplification reaction was carried out in an ABI 7500 Real-Time PCR System (Applied Biosystems, Foster City, CA, USA) with SYBR Green Master Mix (Roche, Basel, Switzerland). The reaction mixture was incubated for 1 min at 95 °C. The amplification program consisted of 40 cycles of denaturation at 95 °C for 15 s and annealing and extension at 60 °C for 45 s. Oligonucleotide primer sequences and annealing temperatures are displayed in Table 1. Transcription levels were normalized against *β-actin*, and each value was the average of three independent extractions. Student’s *t*-test was employed to determine significant changes at 95% confidence level (P <0.05).

**Table 1:** Primers for Realtime RT-PCR. A detailed list of primer sequences, species, genebank numbers, and PCR product lengths used in Realtime RT-PCR.

Gene	Primer sequence	Species	GeneBank accession number	PCR product size (bp)	Temperature (°C)
*Scpp5*	S 5′-TCATTCCCCACACAAGCGTT-3′	*Danio rerio*	NM_001145236.1	200	60.00
	AS 5′-AATGGTGGGTTCACAGGTGG-3′				60.00
*Scpp1*	S 5′-TGACAGCCGACAACACTCAA-3′	*Danio rerio*	NM_001145240.1	196	59.82
	AS 5′-ACGACAACCTTTTCC*TGGCT-* 3′				59.82
*Spp1*	S 5′-ACAGACCACGCCAACAGAAT-3′	*Danio rerio*	NM_001002308.1	188	59.89
	AS 5′-TGATAATGGGACCCAGCGTG-3′				59.82
*Scpp9*	S 5′-CACTTCTGGAGTGAGAAACAGA-3′	*Danio rerio*	NM_001145245.1	216	57.67
	AS 5′-GCTGTCCTATAACCGCAGCA -3′				60.18
*Scpp8*	S 5′-ATGCTTTTTGTTGCGTCTGTCA-3′	*Danio rerio*	NM_001145244.1	187	59.90
	AS 5′-GCCTACGAGTAGGAGGCTGT-3′				60.75
*Odam*	S 5′-CCTGTACAGCTGATGCCCAA-3′	*Danio rerio*	NM_001145243.1	221	60.04
	AS 5′-GGTTCAAACAACGGGAAGCC-3′				59.97
*β-actin*	S 5′-CGAGCTGTCTTCCCATCCA-3′	*Danio rerio*	NM_181601	86	59.10
	AS 5′-TCACCAACGTAGCTGTCTTTCTG-3′				60.80

### Alizarine Red S staining

Adult zebrafish with a standard length of 25 mm (coinciding with approximately 90 dpf) were collected. All fish samples were devitalized, fixed in 4% paraformaldehyde for 24 h, immersed in 0.1% KOH for 48 h, and then macerated with 1%–2% KOH for 8 h to thoroughly remove the residual soft tissue. The samples were then stained with 0.1% Alizarine Red S (Sigma, USA) in 0.5% KOH for 14–20 h, followed by rinsing with sterile water for 12 h; the water was replaced at intervals of one hour. The fifth ceratobranchial arch, which is also called the pharyngeal bone, was carefully dissected and photographed under a stereomicroscope (*n* = 8 for each group).

### Statistical analysis

All data was presented as means ± standard deviation (SD). Comparisons between groups were conducted using Student’s *t*-test performed on Graphpad Prism 8 statistical software Graphpad Prism 8.0.2 (USA). Statistically significant differences were noted when P <0.05.

## Results

### Generation of *dlx3b* mutant zebrafish lines

**Figure 1:**
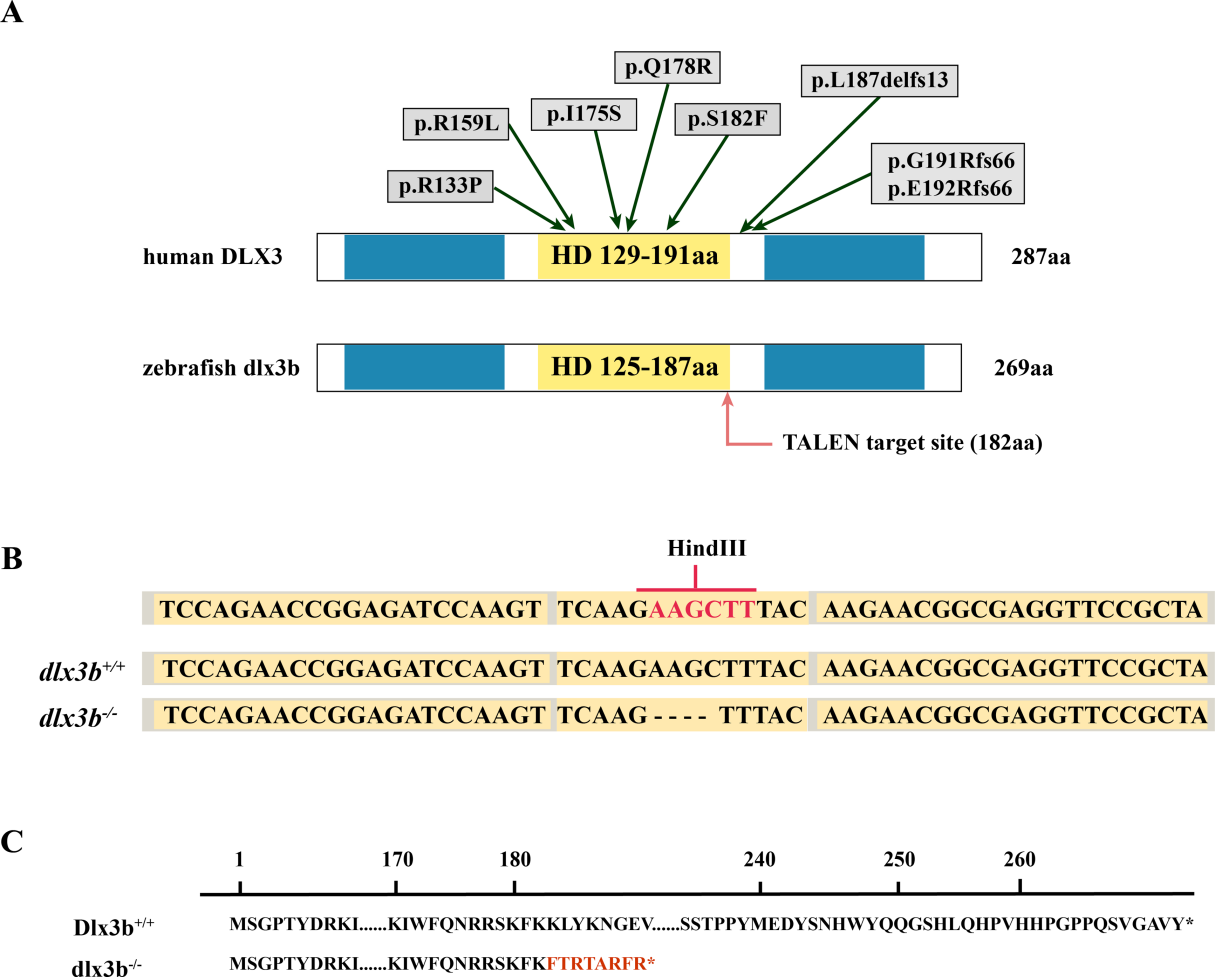
Generation of the mutant *dlx3b* zebrafish line. (A) Structure of the** DLX3 and dlx3b proteins in human and zebrafish. The homeodomain and transactivation domains are marked in yellow and blue, respectively. The green arrows indicate the TDO mutation sites that found in patients. The red arrow indicates the TALEN target site in zebrafish. (B) Sequence of the TALEN target site and mutant site in zebrafish *dlx3b*. TALEN binding sites in the *dlx3b* gene are indicated in gray shading, and the HindIII restriction site is highlighted in red. The sequence of mutant *dlx3b* mRNA is aligned with wild-type *dlx3b* mRNA. (C) Predicted peptide sequences for the wild-type and mutant dlx3b. There is a frameshift mutation at 182 aa, and the subsequent mutant sequence is highlighted in red.

**Figure 2:**
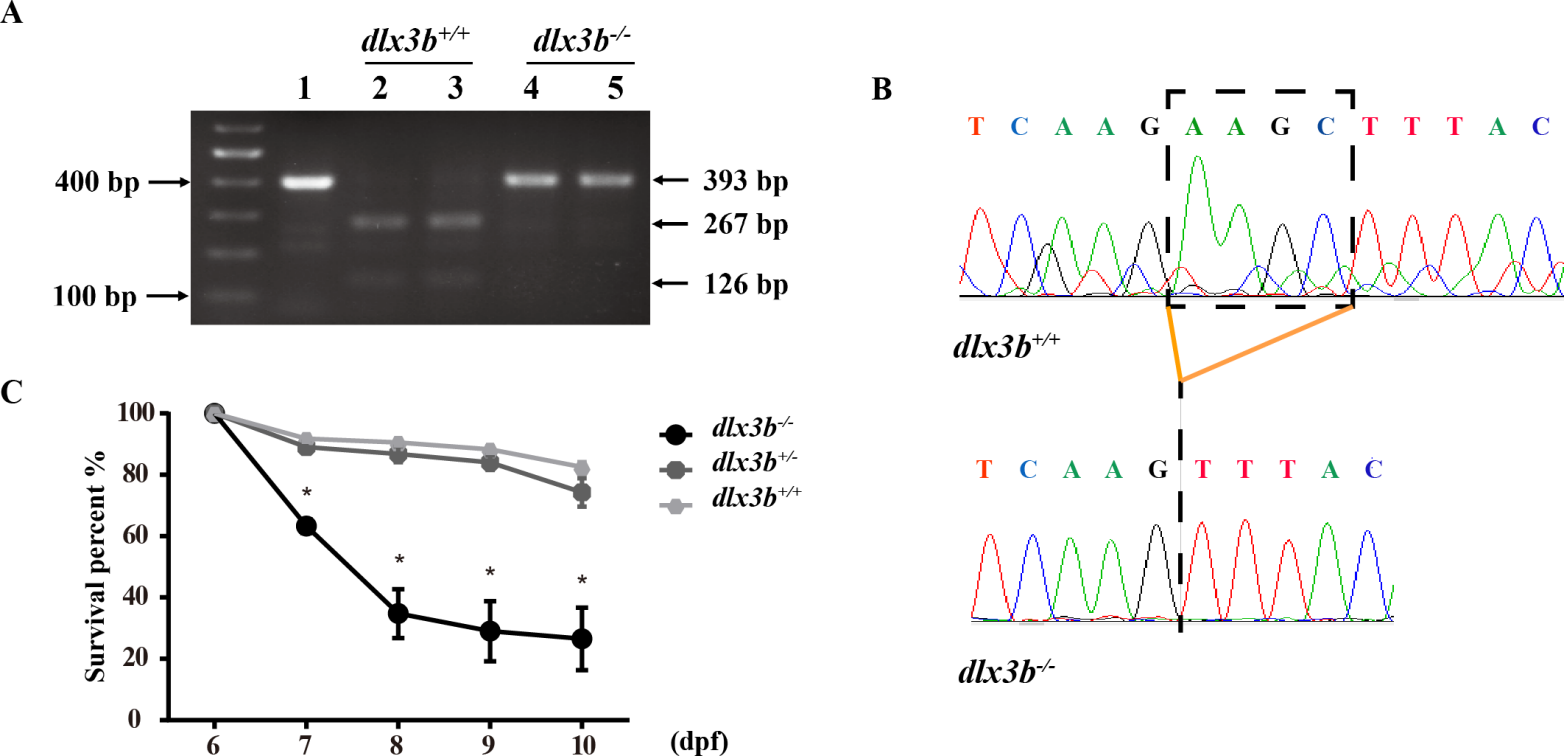
Identification and breeding of the mutant *dlx3b* zebrafish. (A) Genotyping results identifying homozygote (*dlx3b*^−∕−^) mutant and wild-type siblings (*dlx3b*^+∕+^). Genomic DNA was amplified by PCR from the tails of the first-generation (F1) fish and then digested with a diagnostic restriction enzyme (Hind III). The first lane contains the original PCR product (393 bp). Lanes 2 and 3 contain wild-type siblings (*dlx3b*^+∕+^), and lanes 4 and 5 contain homozygotes (*dlx3b*^−∕−^). (B) Sequencing peak map of the DNA sample from the wild-type siblings (*dlx3b*^+∕+^) and mutant (*dlx3b*^−∕−^) zebrafish, respectively. The absence of the four-bp sequence (AAGC) in the mutant zebrafish (*dlx3b*^−∕−^) is clearly marked. (C) Homozygous mutant (*dlx3b*^−∕−^) zebrafish exhibited a lower survival rate compared with that of their wild-type (*dlx3b*^+∕+^) and heterozygous siblings (*dlx3b*^+∕−^) from 6–10 dpf. Within 5 d (6–9 dpf), the survival rate of the *dlx3b*^−∕−^ group decreased from 100% to approximately 35%, while those of the *dlx3b*^+∕+^ group and *dlx3b*^+∕−^ group decreased to 75%–80%.^∗^*P* < 0.05 *vs*. The *dlx3b*^+∕+^ group. *n* = 100 for each group (the starting population).

The TALEN targeting site in the zebrafish *dlx3b* gene model was designed at the 3′-terminal end of the homeodomain (Fig. 1A). By using the TALEN-mediated genome editing method, the predicted *Hind* III restriction enzyme digestion site (AAGCTT) in the *dlx3b* gene was disrupted, resulting in a four-bp deletion mutation from 544 bp to 547 bp (Fig. 1B). This *dlx3b* mutation introduced a frameshift and premature stop codon, shortening the protein sequence to 189 aa in total. Notably, in the mutant dlx3b protein, the 182nd lysine (K) was altered to phenylalanine (F) and followed by 7 novel amino acids (Fig. 1C). During fish development, the tails of 1-month-old zebrafish were collected and pooled after genotyping. Under *Hind* III restriction endonuclease digestion, the intact 513 bp PCR product of *dlx3b*^−∕−^ fish tail could not be cut into 286 bp and 226 bp fragments, which was consistent with the four-bp deletion mutation in the *dlx3b* gene (Fig. 2A). The PCR product was further confirmed the exact mutant sequence in the *dlx3b*^−∕−^ zebrafish model (Fig. 2B). During zebrafish breeding, we found that the survival rate of *dlx3b*^−∕−^ larvae decreased dramatically from 6 to 10 dpf, as compared with that of wild type (*dlx3b*^+∕+^) and heterozygous (*dlx3b*^+∕−^) siblings (Fig. 2C). Still, there were enough mutant fish available for subsequent investigations.

### Curved spine and hypoplasia of vertebrates were observed in *dlx3b*^−∕−^ zebrafish

**Figure 3:**
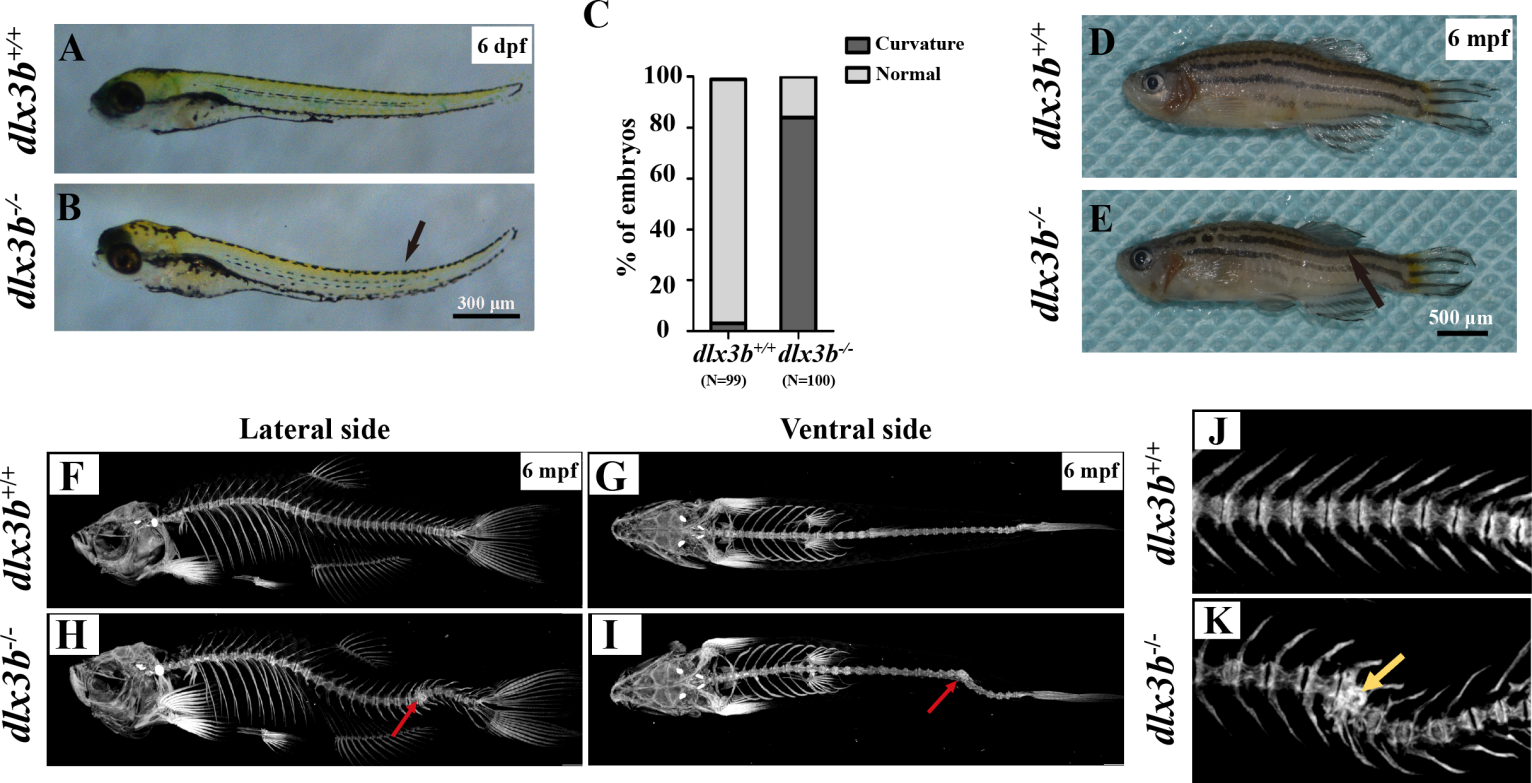
Body curvature of *dlx3b* homozygous mutant zebrafish. (A–B) Body curvature of 6–7 dpf larvae under a stereomicroscope. The body curvature in *dlx3b* ^−∕−^ (B) initially occurred in the early juvenile stages and was mainly limited to the distal end of the tail (black arrow), while the wild type (A) was showed no curvature. Scale bar: 300 µm. (C) Relative frequencies of dorsal curvature phenotypes in *dlx3b*^+∕+^ and *dlx3b* ^−∕−^ embryos. *n* = 100 for each group. (D–E) Mutant adult zebrafish (D) exhibited a severe curvature of the spine compared with wild-type siblings (E), especially in the tail part (black arrow). (F–I) Micro-CT results showing the spine from the lateral side (F & H) and ventral side (G & I). The abdominal and caudal spine of the *dlx3b*^−∕−^ zebrafish developed a marked curvature in both the dorsal–ventral (G&I) and medial–lateral planes (F&H, red arrows). (J–K) Magnified imaging showing vertebral hypoplasia in the *dlx3b*^−∕−^ zebrafish. A radiopaque area was observed in the caudal spine region (K, yellow arrow).

Starting in the early larval stages, a moderate curvature of the spine bone toward the dorsal side was found in *dlx3b*^−∕−^ fish, manifesting in 86 out of 100 fish in the *dlx3b*^−∕−^group as compared to 3 out of 99 in the *dlx3b*^+∕+^ group (Figs. 3A & 3B). At the adult stage (3–6 months), three of the thirty homozygotes exhibited prominent abnormal curvatures in the spine (Fig. 3E), while the wild-type siblings appeared normal (Fig. 3D). The micro-CT revealed that the abdominal and caudal spine bones of *dlx3b*^−∕−^ adult fish exhibited marked curvatures on both the dorsal–ventral (Fig. 3G&I) and medial–lateral (Figs. 3F & 3H) planes. A radiopaque area was shown in the caudal spine region of mutant fish under high magnification (Figs. 3J & 3K).

### Abnormal morphology of tooth enameloid with reduction of mineral content in *dlx3b*^−∕−^zebrafish

**Figure 4:**
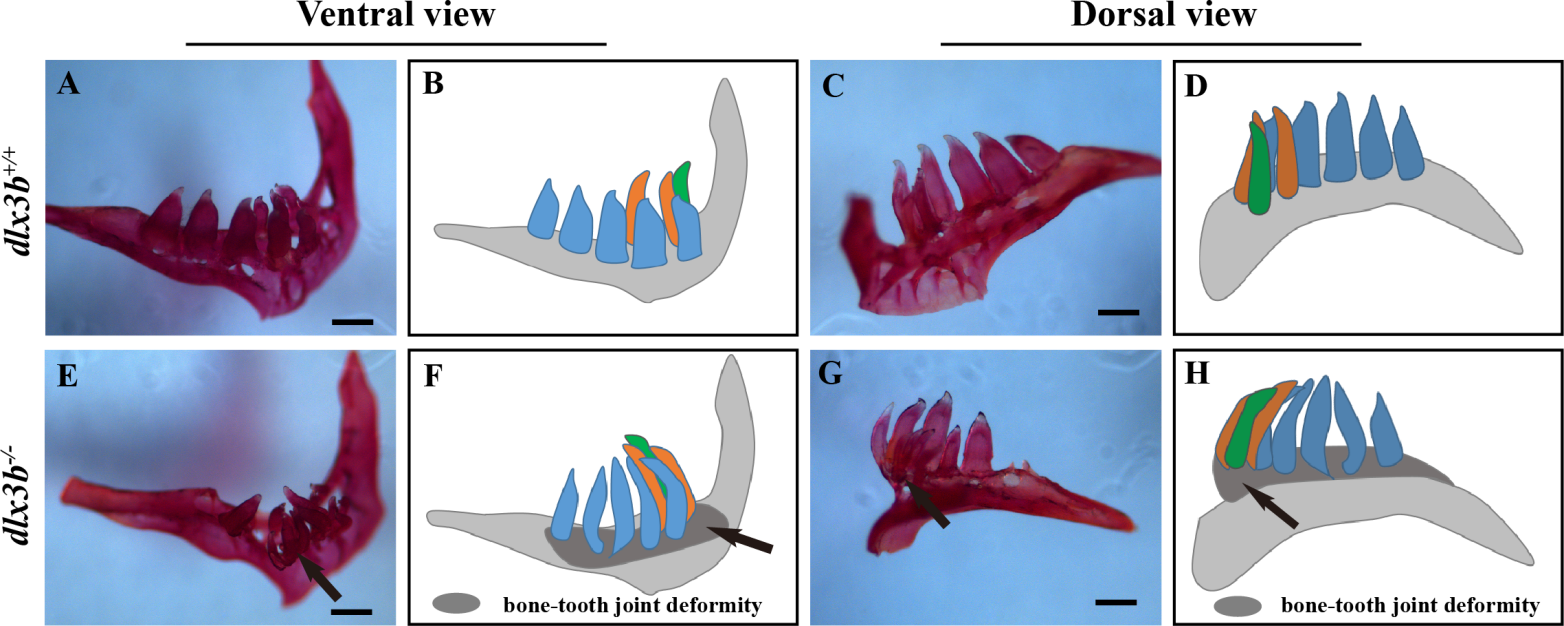
Pharyngeal teeth of homozygous mutants (*dlx3b*^−∕−^) exhibited deformiy. Compared with the pharyngeal teeth of the wild-type (A, C), those of the mutant group exhibited abnormal bending towards the dorsal side (E, G) with the abnormal bone growth of the pharyngeal bone at the bone-tooth joint (E, G, black arrow). (A–D) The pharyngeal dentition of the wild-type sibling; (E–H) the pharyngeal dentition of the *dlx3b*^−∕−^ mutant. (A, E) The pharyngeal dentition viewed from the ventral side. (C, G) The pharyngeal dentition viewed from the dorsal side. (B, D, F, H) Schematic representations of A, C, E, and G, respectively (ventral tooth row, blue; mediodorsal tooth row, ochre; dorsal tooth row, green; replacement teeth not shown; and the abnormal bone growth is depicted as a dark gray region in F and H). Scale bar: 200 µm. *n* = 8 for each group.

Unlike most mammalian teeth, which are supported by alveolar bones, the pharyngeal teeth of zebrafish directly attach to the pharyngeal bone without root. Alizarine Red S staining revealed that six of eight *dlx3b*^−∕−^ zebrafish mutants exhibited a bone-tooth joint deformity in the pharyngeal teeth, especially the ventral teeth (Figs. 4E–4H). None of the examined wild-type siblings exhibited these changes in the pharyngeal bone and dentition (Figs. 4A–4D). Enameloid was visible with the Alizarine Red S staining, but it did not display any visible abnormality at this level.

**Figure 5:**
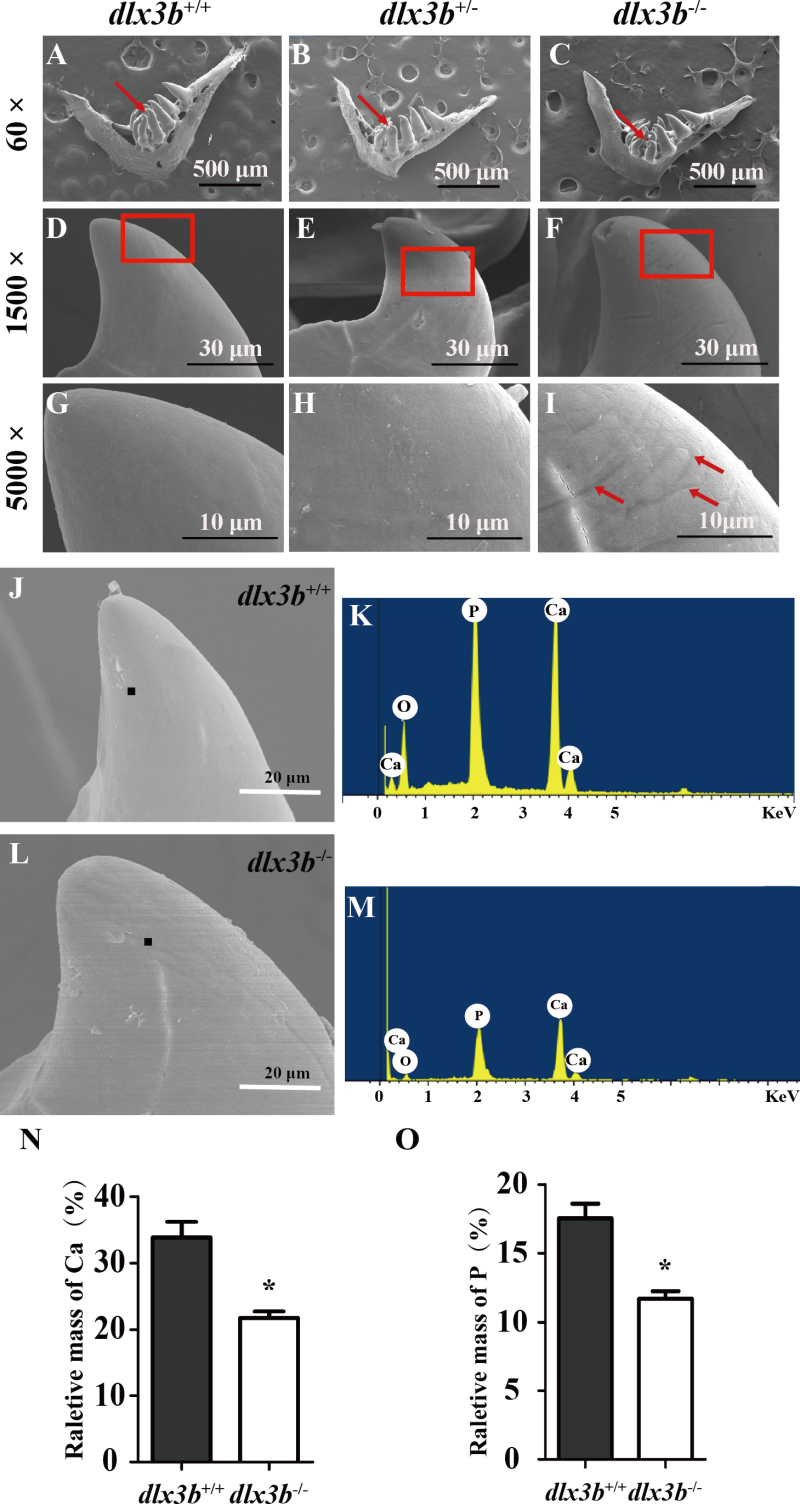
Surfaces of the second and fourth ventral pharyngeal teeth of the *dlx3b* homozygous mutation zebrafish exhibited abnormal pits. (A–I) SEM images showing the surface of the second pharyngeal tooth. The red arrows showed the teeth we studies below in a 60× image (A–C). The fourth ventral pharyngeal teeth (red arrows in A–C) was magnified as images D–F. The red rectangle areas in D–F were then magnified as G–I. The red arrows (I) indicate the pits on the surface of the second ventral pharyngeal tooth of the *dlx3b*^−∕−^ fish. n=10 for each group (J–M) Lower relative mass fractions of mineral elements in the *dlx3b*^−∕−^ mutant. The scanning areas for energy spectrum analysis were selected on the cusp of the pharyngeal teeth showed as images J and L. Energy spectrum of zebrafish pharyngeal teeth was showed as images K and M, mainly including O, Ca, and P. (N–O) The relative mass fraction of Ca (N) and P (O) significantly decreased in the *dlx3b* ^−∕−^ zebrafish compared with those in their wild-type siblings. *n* = 5, P < 0.001.

To elucidate the effects of mutant *dlx3b* on enameloid, we observed the dissected pharyngeal teeth with scanning electron microscopy (SEM). The fourth pharyngeal teeth on the ventral side were accurately identified (Figs. 5A–5I). Under high magnification, eighty percent of *dlx3b*^−∕−^ mutants showed pitting in the enameloid surface of the ventral side compared with the smooth enameloid surface of the wild-type and heterozygous siblings (Fig. 5I). Energy dispersive spectrometry (EDS) was performed on the pitted enameloid regions of *dlx3b*^−∕−^fish to analyze the mineral contents (Figs. 5J–5M). We scanned the same areas in all groups. For each examined tooth, seven areas along the ventral side were chosen and measured. The O, Ca, P, and Au (or Pt) energy spectrum was acquired at each area (Figs. 5J & 5L). The results showed that the relative mass fraction of mineral elements was significantly decreased in *dlx3b*^−∕−^ enameloid, with the calcium content decreasing from 33.84% to 22.73% (Fig. 5N) and phosphorus content decreasing from 17. 52% to 11.71% (Fig. 5O). Specimens were examined by EDS and values were means for *n* = 5 individuals in each group. Nearly ninety percent of *dlx3b*^−∕−^ zebrafish showed a decrease in calcium and phosphorus content.

### SCPP genes were downregulated in *dlx3b* mutants

**Figure 6:**
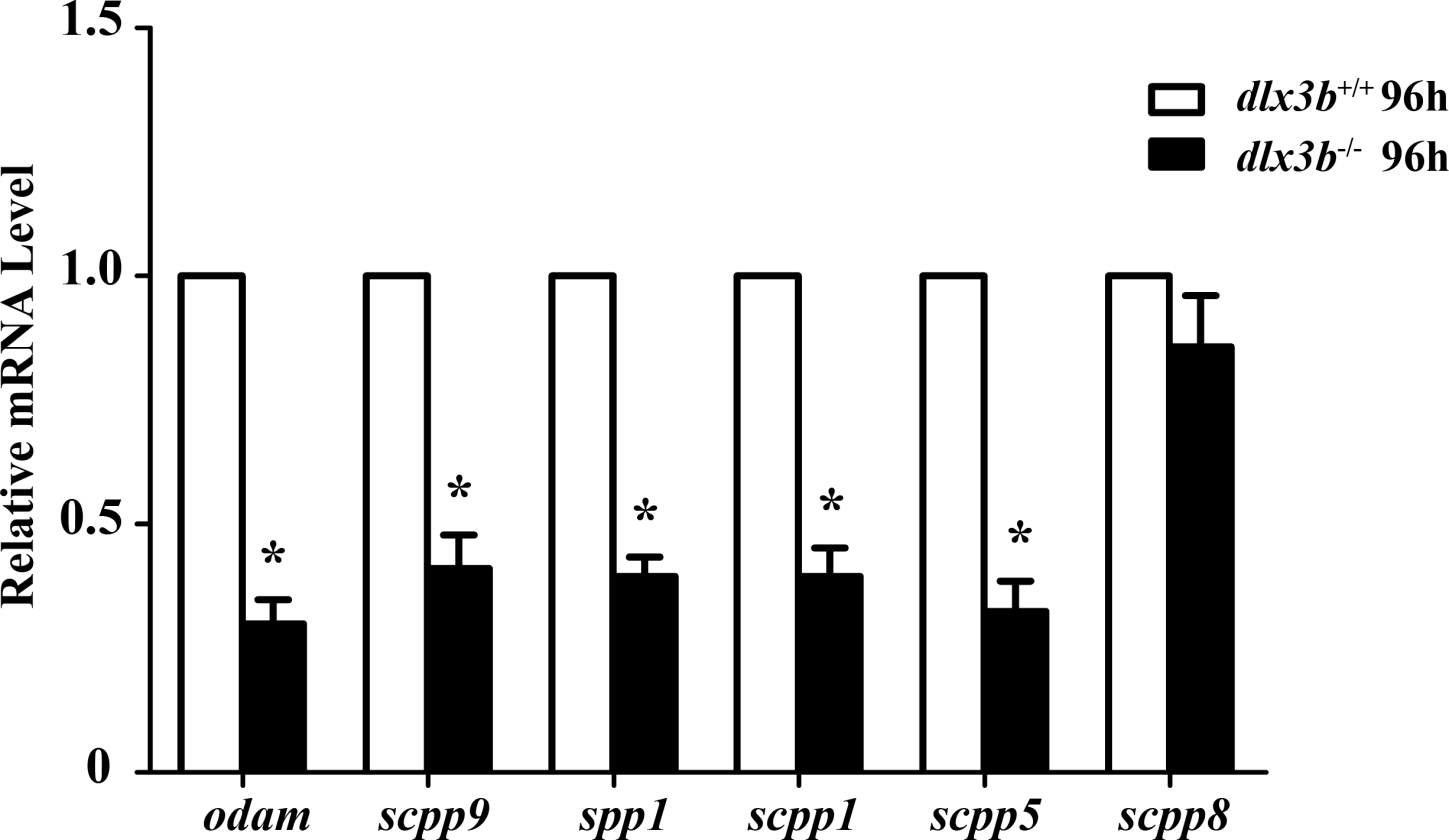
Down regulation of *odam, scpp9, spp1* and *scpp5* in *dlx3b*^−∕−^ fish. Real-time RT-PCR was used to determine the expression levels of mineralized-related genes using RNA samples extracted from zebrafish larvae at 96 hpf. The expression level of *dlx3b*^+∕+^ was set at 1 as a control, and the fold changes in *dlx3b*^−∕−^ were calculated relative to *dlx3b*^+∕+^.^∗^ P < 0.05 vs. the control. * dlx3b*^+∕+^, wild type siblings. *dlx3b*^−∕−^, mutant larvae.

The SCPP family is a group of tandemly duplicated genes involved in the mineralization of hard tissues, including bones and teeth. The principal enamel and dentin protein-related genes in mammals, such as *Amelx*, *Enam*, *Odam* and *Dspp*, all belong to the SCPP family. In *dlx3b* mutant larvae zebrafish, the expression of several SCPP genes, including *odam*, *scpp9*, *spp1*, *scpp1*, and *scpp5*, was significantly downregulated at 96 hpf compared with that in the wild-type siblings (Fig. 6, *n* = 3 independent extractions of 8 pooled individuals each). Among these SCPP genes, *odam* and *scpp9* are mainly involved in the mineralization process of zebrafish enameloid, while *spp1*, *scpp1*, and *scpp5* are closely related to dentin and bone development (Kawasaki, 2009).

## Discussion

Human DLX3 protein has three main domains: the N- and C- terminus transactivation domains, and a central homeodomain that interacts directly with DNA in a sequence-specific way and regulates the expression of target genes (Beanan & Sargent, 2000). Zebrafish *dlx3b* and human *DLX3* shared 78% and 67% conservation in transcripts and proteins, respectively, with the homeodomain sequences showing up to 98.33% conservation. Among the previously reported individuals with TDO, all DLX3 mutations are found to affect residues within, or adjacent to, the homeodomain, indicating the importance of this region in the pathogenesis of TDO. As for the *dlx3b* mutant fish described in our study, the *in situ* mutant site (c.544_547delAAGC) was just within the C- terminus of the homeodomain, introducing a frameshift and premature stop codon in the protein sequence. The *dlx3b*^−∕−^ phenotype of the zebrafish model in this study revealed the functions of *dlx3b* in tooth mineralization and bone formation. Although the survival rate of *dlx3b* mutant fish was considerably decreased, enough mutant fish were viable for morphological and genetic analysis.

As a polyphyodont organism, zebrafish replace their dentition continuously throughout their life. Normally, the zebrafish pharyngeal teeth are attached to the fifth branchial arch (also called the pharyngeal bone) and do not have tooth root encircled by periodontal tissues (Jackman & Stock, 2006). Nevertheless, the development and structure of the crown part of zebrafish’s teeth are very similar to those of mammalian teeth (Arnold et al., 2008). The expression of zebrafish *dlx3b* and other *dlx* family genes was reported in 2006, and the *dlx3b* and *dlx4b* genes were found to be mainly expressed in the dental epithelium (Borday-Birraux et al., 2006; Verreijdt et al., 2006). In zebrafish, simultaneous injection with morpholinos against *dlx2a*, *dlx2b*, *dlx3b,* and *dlx5a* has been reported to cause smaller and misshapen teeth (Jackman & Stock, 2006). We found, however, that the size and general contour of the pharyngeal teeth in the *dlx3b* mutant fish seemed normal. Under high magnification, we found linear pitted defects of the enameloid surface in *dlx3b*^−∕−^ fish, and the mineral content of Ca and P was decreased. The relatively milder tooth defects of *dlx3b*^−∕−^ fish as compared to TDO patients might be explained by the possible redundant function of the *dlx3b* and *dlx4b* genes, as has already been reported concerning their role in optic and olfactory placode development (Solomon & Fritz, 2002; Kaji & Artinger, 2004). Auto- and cross-regulatory control of *dlx3b* and *dlx4b* expression might also occur in zebrafish tooth development. Moreover, the complex epigenetics regulation by environmental factors reported in mammalian studies might exacerbate the enamel defects in human TDO disease.

Skeletal abnormalities within cranial bones have been reported to be associated with mammalian DLX3 mutants, including a lack of mastoid pneumatization, increased thickness of the cranial bones, increased bone density, and a shortened mandibular ramus. Other sites like the radius, ulna and spine bones were also reported to have elevated bone density in TDO patients (Li et al., 2014; Hofstetter et al., 2017). A mixed injection of *dlx3b*, *dlx4b*, and *dlx5a* morpholinos in zebrafish caused dramatic facial skeletal defects, including joint loss, bone fusion, and ectopic processes (Talbot, Johnson & Kimmel, 2010). In this study, the pharyngeal bone deformity at the bone-tooth joint in *dlx3b*^−∕−^ fish was detected by Alizarine Red S staining. Moreover, both the juvenile and adult mutant fish spine showed an abnormal curvature in the abdominal and caudal regions, although no significant change was detected in adult bone density (Fig. S1). The spine curvatures were seen in *dlx3b*^−∕−^ fish at both the juvenile and adult stages, ahead of the age at which senescent bending of the spine might occur in aged zebrafish (around 52 months) (Gerhard et al., 2002; Jian et al., 2018). The bone density changes found in individuals with TDO were not detected in the pharyngeal bone or spine of our zebrafish model, possibly due to the differences in function of the mutated protein or the evolutionary differences existing between different vertebrate skeletal systems.

Furthermore, we analyzed the expression of mineralization-related genes in the mutant fish and found that several SCPP genes (*scpp9, scpp5, odam, scpp1, spp1*) were significantly downregulated, indicating that these genes were involved in the molecular mechanisms responsible for *dlx3b* function in zebrafish. Previous *in vitro* studies suggested that in ameloblast cells, mutant *Dlx3* could directly downregulate the expression of three essential SCPP genes, *Amelx*, *Enam* and *Odam* (Zhang et al., 2015; Yan et al., 2017). Similarly, in this study, the downregulated expression of *odam* was found in *dlx3b*^−∕−^ fish, together with other SCPP genes (*scpp9*, *scpp5, spp1*, and *scpp1*). These results confirmed the conserved function of the *dlx3b* gene in regulating SCPP genes during the evolution of dental tissues and suggested that the defects in enamel and enameloid may be attributed to the disrupted mineralization process mediated by SCPP proteins. It was reported that a Dlx3 deletion in mouse dental epithelium significantly downregulated several ion transporters and carbonic anhydrases, but not the SCPP family genes (Duverger et al., 2016; Duverger & Morasso, 2018). This demonstrates the different *in vivo* impacts of Dlx3 deletion versus dlx3 mutation, which might be related to species differences between mice and zebrafish.

## Conclusion

In conclusion, our study demonstrates the effects of *dlx3b* on tooth mineralization and bone formation. By use of *dlx3b* mutant zebrafish, we illustrate that the four-bp deletion mutation from 544 bp to 547 bp in *dlx3b* resulted in decreased enameloid mineralization and curved spine, and certain SCPP genes were down-regulated. The *dlx3b*^−∕−^ zebrafish model is helpful for understanding the function of the *dlx3* gene, although the development and structure of zebrafish teeth are not completely mimic of mammals. Further study is needed to deepen the study of function and pathogenesis of *DLX3* gene.

##  Supplemental Information

10.7717/peerj.8515/supp-1 Figure S1 Relative bone density from CT data BMD from CT scan data was showed with three ROIs (Regions-of-interests) The data showed no significant change between *dlx3b*^+∕+^ *and dlx3b*^−∕−^.

10.7717/peerj.8515/supp-2 Figure S2 The CT scan image was presented from caudal view, an anterior-posterior axis exactly The fifth pharyngeal jaw of *dlx3b*^+∕+^ (A) and *dlx3b*^−∕−^ (B) were clearly showed.

10.7717/peerj.8515/supp-3 Figure S3 The tooth size measurement based on the SEM data shows that there are no significant difference of tooth size

10.7717/peerj.8515/supp-4 Figure S4 The bone density showed no significant difference presented as whole bone (A), craniofacial bone (B) and body bone (C)

10.7717/peerj.8515/supp-5 Supplemental Information 1 Generation of dlx3b mutant zebrafish lines

10.7717/peerj.8515/supp-6 Supplemental Information 2 Identifying and breeding of the mutant *dlx3b* zebrafish

10.7717/peerj.8515/supp-7 Supplemental Information 3 Body curvature of *dlx3b* homozygous mutant zebrafish

10.7717/peerj.8515/supp-8 Supplemental Information 4 Pharyngeal teeth of homozygous mutants (*dlx3b*^−∕−^) exhibited gryposis toward the dorsal side

10.7717/peerj.8515/supp-9 Supplemental Information 5 Surfaces of the second and fourth ventral pharyngeal teeth of the *dlx3b* homozygous mutation zebrafish exhibited abnormal dent

10.7717/peerj.8515/supp-10 Supplemental Information 6 The expression of *odam, scpp9*,* spp1, scpp1*, and* scpp5* was down-regulated in the *dlx3b* mutant zebrafish

10.7717/peerj.8515/supp-11 Supplemental Information 7 Relative bone density from CT data

10.7717/peerj.8515/supp-12 Supplemental Information 8 The CT scan image of zebrafish head was presented from caudal view

10.7717/peerj.8515/supp-13 Supplemental Information 9 Raw data for tooth size

10.7717/peerj.8515/supp-14 Supplemental Information 10 Supplemental data for bone density

## Additional Information and Declarations

### Data Availability

The following information was supplied regarding data availability: The raw measurements are available in the Supplementary Files.
