# Supplementary figures and images for: Mutant dlx3b disturbs normal tooth mineralization and bone formation in zebrafish
Source: PeerJ. 2020 Feb 19;8:e8515. doi: 10.7717/peerj.8515 (PMC7035872; doi:10.7717/peerj.8515)

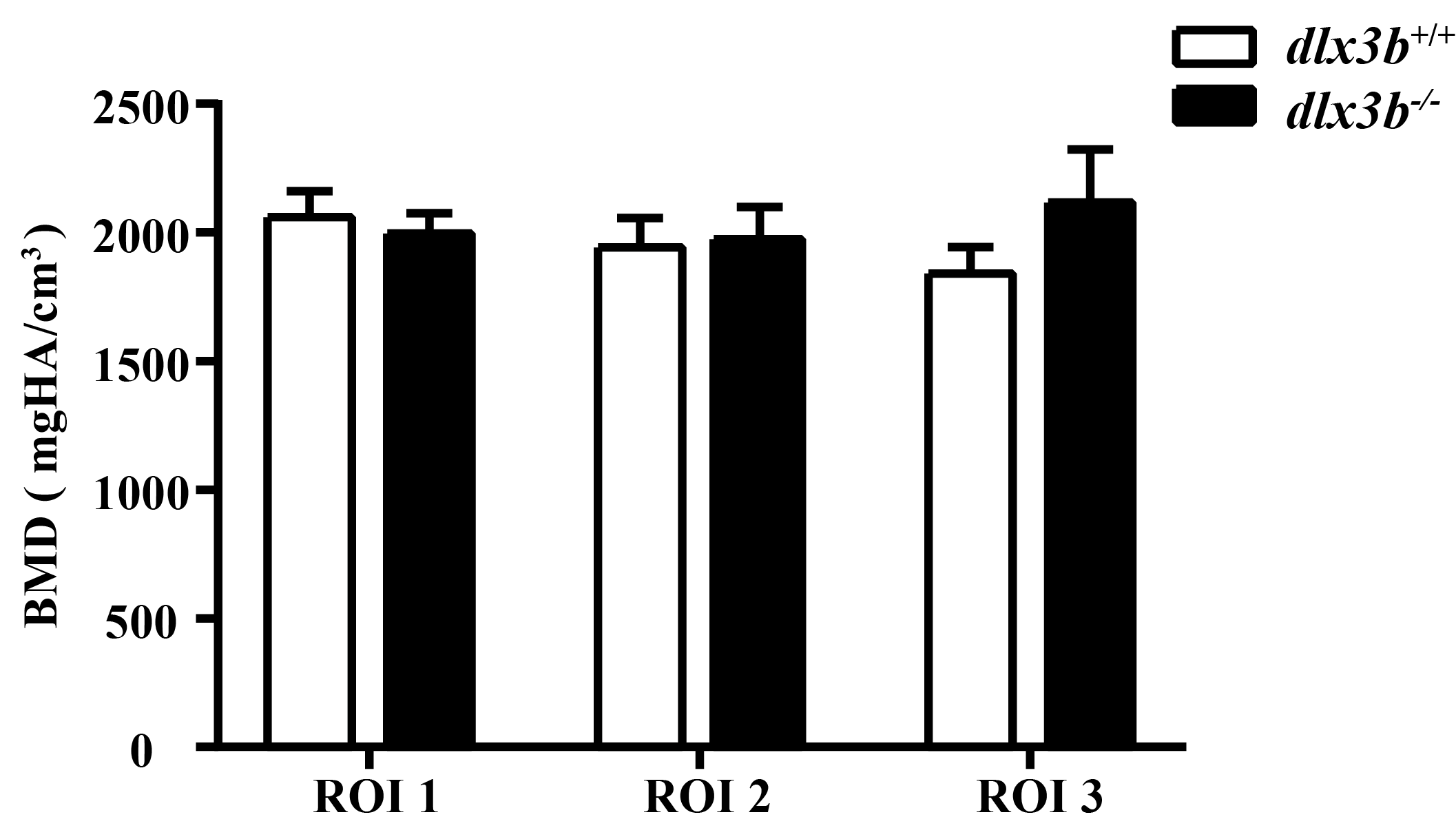

Supplement: Figure S1 — BMD from CT scan data was showed with three ROIs (Regions-of-interests) The data showed no significant change between dlx3b+∕+ and dlx3b−∕−. [file peerj-08-8515-s001.png]

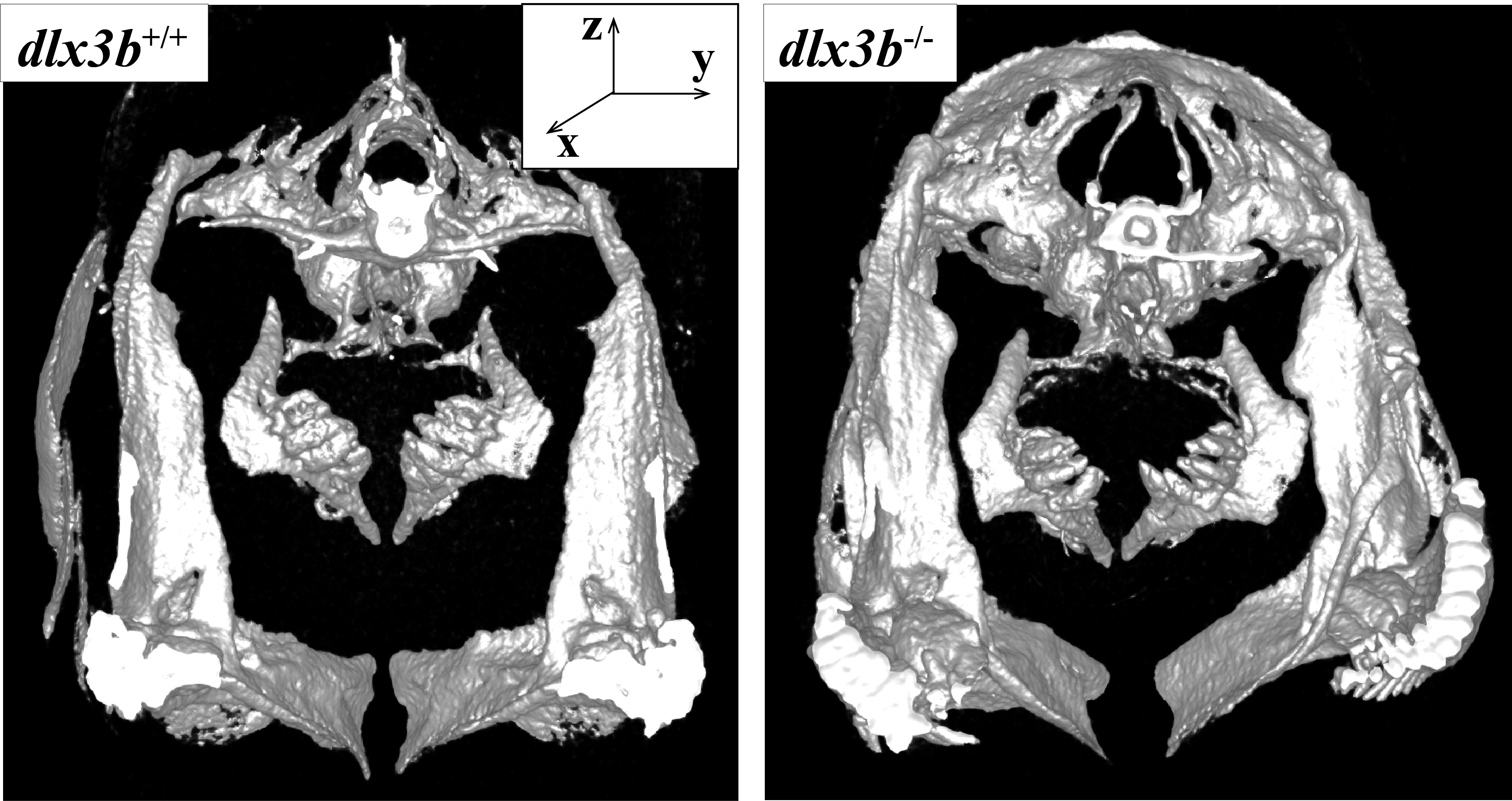

Supplement: Figure S2 — The fifth pharyngeal jaw of dlx3b+∕+ (A) and dlx3b−∕− (B) were clearly showed. [file peerj-08-8515-s002.png]

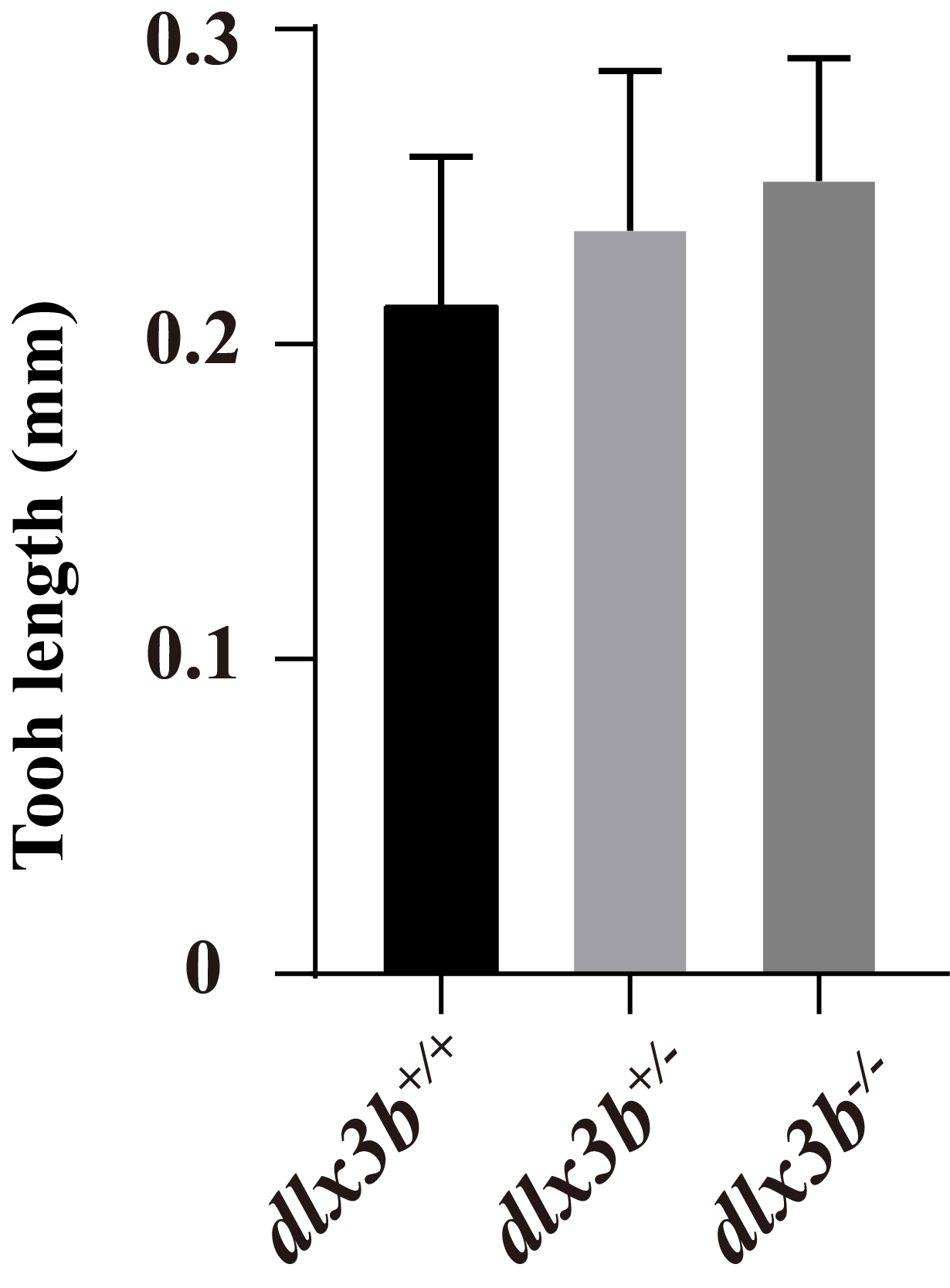

Supplement: Figure S3 [file peerj-08-8515-s003.png]

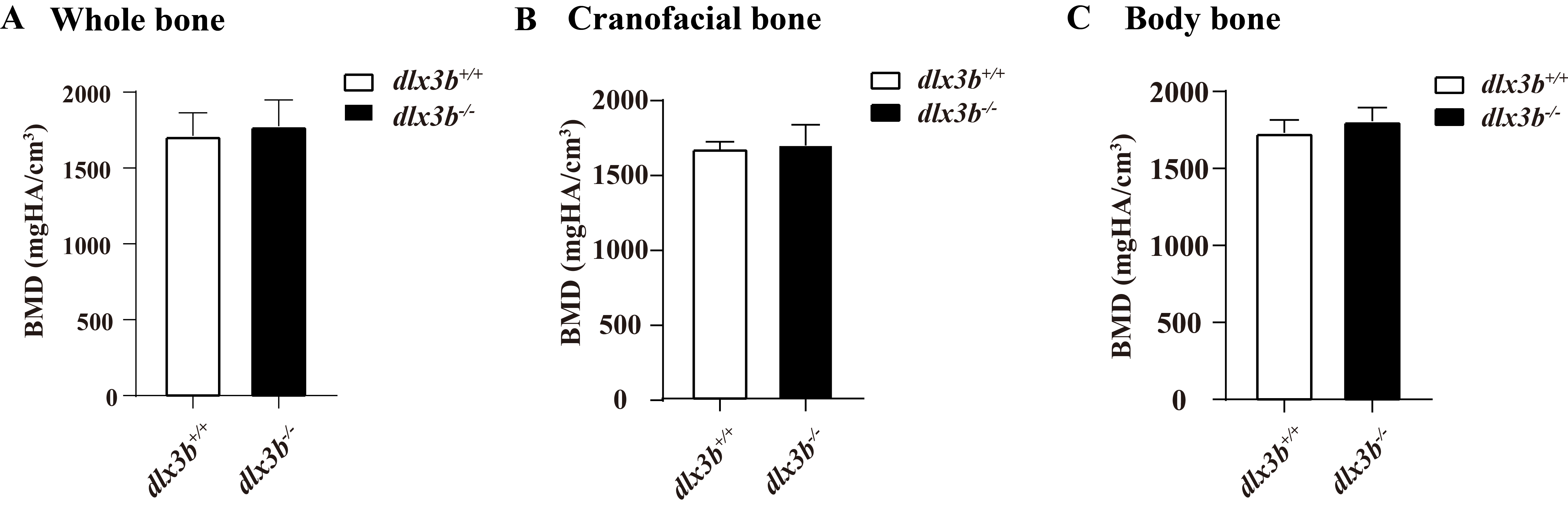

Supplement: Figure S4 [file peerj-08-8515-s004.png]
